# Plasma Exosomal Mir-423-5p Is Involved in the Occurrence and Development of Bicuspid Aortopathy *via* TGF-β/SMAD2 Pathway

**DOI:** 10.3389/fphys.2021.759035

**Published:** 2021-12-10

**Authors:** Hongqiang Zhang, Dingqian Liu, Shichao Zhu, Fanshun Wang, Xiaoning Sun, Shouguo Yang, Chunsheng Wang

**Affiliations:** Department of Cardiac Surgery, Zhongshan Hospital, Fudan University, Shanghai, China

**Keywords:** exosomal miRNA, bicuspid aortic valve, bicuspid aortopathy, TGF-β signaling, SMAD2

## Abstract

Objectives: Patients with bicuspid aortic valve (BAV) are at increased risk for ascending aortic dilation (AAD). Our study was aimed at systemically analyzing the expression profile and mechanism of circulating plasma exosomal microRNAs (miRNAs) related to BAV and AAD.

Methods: We isolated plasma exosomes from BAV patients (*n*=19), BAV patients with AAD (BAVAD, *n*=26), and healthy tricuspid aortic valve individuals with low cardiovascular risk (TAVnon, *n*=16). We applied a small RNA sequencing approach to identify the specific plasma exosomal miRNAs associated with BAV (*n*=8) and BAVAD (*n*=10) patients compared with healthy TAVnon (*n*=6) individuals. The candidate differentially expressed (DE) miRNAs were selected and validated by RT-qPCR in the remaining samples. GO and KEGG pathway enrichment analyses were performed to illustrate the functions of target genes. Western blot analysis and luciferase reporter assay were conducted in human aortic vascular smooth muscle cells (VSMCs) to verify the results of target gene prediction *in vitro*.

**Results:** The expression levels of three up-regulated (miR-151a-3p, miR-423-5p, and miR-361-3p) and two down-regulated (miR-16-5p and miR-15a-5p) exosomal miRNAs were significantly altered in BAV disease. Additionally, miR-423-5p could be functionally involved in the occurrence and development of BAV and its complication BAVAD by regulating TGF-β signaling. miR-423-5p could target to SMAD2 and decreased the protein levels of SMAD2 and P-SMAD2.

Conclusion: Plasma exosomal miR-423-5p regulated TGF-β signaling by targeting SMAD2, thus exerting functions in the occurrence and development of BAV disease and its complication bicuspid aortopathy.

## Introduction

Bicuspid aortic valve (BAV), the most common cardiac congenital abnormality, occurs in 0.5 to 2% of the population, and the ratio of men to women is about 3 to 1 (Siu and Silversides, 2010; Liu et al., 2019). Additionally, approximately 50–70% of BAV individuals develop bicuspid aortopathy and subsequent premature cardiovascular complications, such as ascending aortic dilation (AAD), ascending aortic aneurysm (AAA) or aortic dissection (AD; Verma and Siu, 2014). These disorders were potentially fatal, which lead to the increased risk of aortic rupture (Fedak et al., 2002; Tadros et al., 2009). Among these complications, BAV with AAD, or BAVAD for short, arouses our interest, but the mechanism related to BAVAD development is still vague.

Over the past decades, much effort has been made in investigating the pathogenesis of BAV. Some indicated that the development of bicuspid aortopathy was attributed to genetic and hemodynamic factors (Verma and Siu, 2014; Liu et al., 2019). Others believed that BAV patients were at increased risk for bicuspid aortopathy and aneurysm associated with extracellular matrix (ECM) degradation with a deficiency of fibrillins (FBNs) and increased matrix metalloproteinases (MMPs; Verma and Siu, 2014), and most bicuspid aortopathy patients showed defective FBN1 and increased MMP (especially MMP2 and 9) activities in the tissue of the dilated aorta (Wang et al., 2021).

Recent studies have focused on the role of microRNAs (miRNAs) in the occurrence and development of BAV and its complications by regulating the expression of critical biomarkers. miRNAs are a type of non-coding RNAs with 20–25 nucleotides in length, which can directly bind to their downstream messenger RNAs (mRNAs) to suppress their expression (Treiber et al., 2019). For example, progressive aortic dilation was reported to be regulated by miR-17-associated miRNAs through the down-regulation of tissue inhibitors of metalloproteinases (TIMPs; Wu et al., 2016). The expression profile of circulating miRNAs associated with BAV and aortic complications was evaluated using a miRNome-wide microarray approach (Martínez-Micaelo et al., 2017). Evidence suggested that a miR-200-dependent process of endothelial/epithelial mesenchymal transition was a plausible mechanism rendering BAVAD more prone to aneurysm development (Maleki et al., 2019).

Exosomes are small extracellular vesicles (EVs) enclosed in lipid bilayer membranes and participate in cell-to-cell communication in normal or pathological states (Bei et al., 2017). Exosomes contain natural cargo molecules, including small non-coding RNAs, mRNAs, and proteins, and transfer these cargoes to neighboring cells or distant cells through circulation (Sun et al., 2018). Large numbers of miRNAs have been identified to be related to BAV and BAVAD; however, the mechanism of circulating miRNAs in plasma-derived exosomes and their mechanisms in the development of BAV and related complications remain unclear. Our study focused on the miRNAs derived from plasma exosomes to find the biomarkers associated with the occurrence and development of BAV or BAVAD, providing guidance for the diagnosis and treatment of these diseases.

## Materials and Methods

### Patients and Plasma Samples

The patients for this study belonged to a cohort of patients who underwent open-heart surgery at Zhongshan Hospital, Shanghai, China, for BAV or BAVAD disease. Patients were classified according to aortic valve cuspidity and dilation. Aortic diameters of >45mm and<40mm were classified as dilated (BAVAD) and non-dilated (BAV; Erbel et al., 2014). Patients with impaired systolic ventricular function, significant coronary artery disease, or aortic dissection were excluded from the study. Healthy individuals with tricuspid aortic valve and low cardiovascular risk (TAVnon) were selected as controls. In total, 19 BAV patients, 26 BAVAD patients, and 16 healthy TAVnon individuals were included. The clinical and demographic characteristics of patients for small RNA sequencing and RT-qPCR validation are shown in Tables 1, 2, respectively. The plasma samples for this study were gathered in vacuum blood tubes with anticoagulant before operation and handled within 1h after collection as previously described (Witwer et al., 2013). This study was approved by the Human Research Ethics Committee of Zhongshan Hospital (B2018-285R). Written informed consent was obtained from all patients according to the Declaration of Helsinki.

**Table 1:** Baseline characteristics.

Variables	BAV (*n* =8)	BAVAD (*n* =10)	TAVnon (*n* =6)	value of *p*
Age (years)	56.67±10.64	56.4±12.42	50.5±3.69	0.4788
Gender (M/F)	7/2	5/4	4/2	0.607
Height (cm)	160.44±6.53	162.5±5.39	165±5.22	0.359
Weight (kg)	63.89±13.37	61.6±9.44	63.33±10.33	0.9018
BSA (m^2^)	1.76±0.2	1.75±0.15	1.8±0.15	0.8426
Hypertension (y/n)	3/5	3/7	2/4	0.945
Hyperlipidemia (y/n)	1/7	0/10	0/6	0.352
Sinus Dilation	1/7	2/8	0/6	0.504
AAO (mm)	37.89±3.21	49.7±2.41	39.89±2.55	<^**,***^
PG (mean, mmHg)	56.78±17.25	36.5±16.14	54.22±15.55	0.0328^*^
PSV (m/s)	4.86±0.71	3.9±0.86	5.01±0.83	0.0195^*^
Aortic Stenosis	8/0	10/0	0/6	0.00^**,***^
Aortic regurgitation	3/5	2/8	0/6	0.231

* *p<0.05;*

** *p<0.01*;

*** *p<0.001*.

*BSA, body surface area, AAO, diameter of ascending aorta, PG, pressure gradient, PSV, peak systolic velocity*.

**Table 2:** Baseline characteristics of validation groups.

Variables	BAV (*n* =11)	BAVAD (*n* =16)	TAVnon (*n* =10)	value of *p*
Age (years)	56.33±11.37	54±7.41	49.43±9.21	0.233
Gender (M/F)	6/5	10/6	7/3	0.766
Height (cm)	160.56±6.48	164.31±5.81	162.5±6.33	0.3082
Weight (kg)	62.28±9.67	67.97±11.9	65.22±9.28	0.3999
BSA (m^2^)	1.74±0.15	1.84±0.17	1.86±0.16	0.1871
Hypertension (y/n)	5/6	6/10	4/6	0.917
Hyperlipidemia (y/n)	1/10	0/16	0/10	0.297
Sinus Dilation	3/8	4/12	0/10	0.2
AAO (mm)	39.89±3.41	49.19±2.7	40.09±2.99	<^**,***^
PG (mean, mmHg)	52.22±18.77	41.69±11.77	56.22±13.32	0.041^*^
PSV (m/s)	4.64±0.69	4.17±0.57	4.78±0.7	0.0494^*^
Aortic Stenosis	11/0	16/0	0/10	0.000^**,***^
Aortic regurgitation	3/8	5/11	0	

* *p<0.05*;

** *p<0.01*;

*** *p<0.001*.

*BSA, body surface area, AAO, diameter of ascending aorta, PG, pressure gradient, PSV, peak systolic velocity*.

### Exosome Isolation and Identification

The exosomes were isolated by using ExoQuick Exosome Isolation Kit (System Biosciences, Mountain View, CA, United States) according to the manufacturer’s instructions. NanoSight LM10 System was adopted to measure the rate of Brownian motion for calculating nanoparticle concentrations and analyzing size distribution through a particle-tracking and fast video capture software. Besides, exosomes to be examined by transmission electron microscopy (TEM) were suspended in dioxyuranium acetate, applied to copper grids, precipitated, dried, and imaged. The EV-specific marker CD63 (CD63 antibody, 1:1000, SBI, China) was also detected by western blot analysis to identify the exosomes.

### RNA Isolation, Small RNA Library Construction, and Sequencing

Total RNA was isolated from plasma exosomes of each group with TRIzol reagent (Invitrogen, Carlsbad, CA, United States). Then, the quantity and purity of total RNA were determined by NanoDrop spectrophotometer (Thermo Fisher Scientific, Waltham, MA, United States). Small RNA libraries were established using NEBNext Multiplex Small RNA Library Prep Kit for Illumina (New England Biolabs, Ipswich, MA, United States) according to the manufacturer’s protocol. Finally, small RNA sequencing was performed on Hiseq 2,500 System (Illumina, San Diego, CA, United States).

### Data Filtering and Mapping

Raw transcriptome sequencing data were filtered and optimized with FastQC software^1^ by discarding low-quality reads and short reads (< 15 nucleotides). The clean reads of small RNAs were then matched to miRBase database^2^ to identify known miRNAs (20–25 nucleotides), and the unmapped reads were further aligned to piRNAcluster database.^3^ The reads which could be compared to piRNAcluster data were considered as potential piwi-interacting RNAs (piRNAs; 24–33 nucleotides) and then mapped to the data from national center for biotechnology information (NCBI)^4^ to validate known piRNAs. Additionally, the unmapped reads were further compared with known ribosomal RNAs (rRNAs; 12–43 nucleotides) from NCBI database to get the rRNA-derived piRNAs. Subsequently, Genomic tRNA database (GtRNAdb)^5^ was used to analyze the unmapped reads. The reads mapped to GtRNAdb were then presented to tRFdb^6^ to identify transfer RNA (tRNA) derived fragments (tRFs). The reads unmapped to GtRNAdb were then assembled into small nucleolar RNA (snoRNA) and small nuclear RNA (snRNA) sequences from Rfam^7^ (Kalvari et al., 2018).

### Screening of Differentially Expressed miRNAs and Prediction of Their Target Genes

Differentially expressed (DE) miRNAs from each group of plasma exosomes were screened with Ebseq 2.0 packages. Cytoscape software (version 3.5.1) was applied to construct the integrated miRNA/mRNA network (Shannon et al., 2003).

### Gene Ontology and Pathway Analysis of Predicted Target Genes

The biological functions of the screened target genes were then evaluated by Gene Ontology analysis (Hulsegge et al., 2009). Pathway enrichment analysis of the screened target genes was performed according to the Kyoto Encyclopedia of Genes and Genomes to explore the biological pathways in which the co-expressed genes were potentially to be involved (Minoru and Susumu, 2000). The cutoff threshold was set to value of *p*<0.05. Venn diagrams were depicted to analyze the DE miRNAs with the same upward or downward trend comparing the exosome samples from BAVAD or BAV group and TAVnon group, or BAVAD and BAV groups.

### Real-Time Quantitative PCR

RevertAid First Strand cDNA Synthesis Kit (Thermo Fisher Scientific) was used to reversely transcribe total RNAs into complementary DNAs (cDNAs) according to the manufacturer’s instructions. qPCR was conducted on the ABI Q6 detection system (Applied Biosystems Inc., Foster City, CA, United States) to measure the relative abundances of miRNAs. The sequences of primers (Shanghai Yingbio Technology, Co., Ltd., Shanghai, China) are shown in Supplementary Materials S1. U6 was used as the internal control.

### Cell Culture and Transfection

Human aortic vascular smooth muscle cells (VSMCs; ScienCell Research Laboratories, Carlsbad, CA, United States) were resuscitated and cultured in smooth muscle cell growth medium (Gibco, Grand Island, NY, United States) according to the requirements. miR-423-5p mimics and negative control (NC) were purchased from Genechem (Shanghai, China). Cell transfection was conducted using Lipofectamine 2000 (Invitrogen).

### Western Blot Analysis

According to whether miR-423-5p mimics or NC were transfected, VSMCs were divided into three groups: blank control group (Blank), negative control group (NC) and experimental group (miR-423-5p mimics). After incubation with primary antibodies (Abcam) against SMAD2, P-SMAD2, SMAD3, P-SMAD3 or SMAD4 and horseradish peroxidase (HRP)-conjugated secondary antibody (Abcam), protein bands were detected using ECL detection system (Amersham Pharmacia, Piscataway, NJ, United States). GAPDH was used as internal control. Each group was repeated three times, and the average value was the experimental results.

### Luciferase Reporter Assay

The recombinant luciferase plasmids using pMIR vectors (Promega, Madison, WI, United States) harboring SMAD2 3' untranslated region (3'UTR) and SMAD2-mut 3'UTR with mutant binding regions for miR-423-5p were constructed. The above plasmids were transfected into human aortic VSMCs, which were divided into five groups: pMIR vector, pMIR-SMAD2-plasmid + NC, pMIR-SMAD2-plasmid + miR-423-5p mimics, pMIR-SMAD2-mut-plasmid + NC, and pMIR-SMAD2-mut-plasmid + miR-423-5p mimics. Relative luciferase activity was examined using Dual-Luciferase Reporter Assay System (Promega) as the ratio of firefly/Renilla luciferase activity. The enzymatic activities of firefly and control Renilla luciferases were sequentially measured in every single sample using Dual-Luciferase Reporter Assay System (Promega) on addition of Stop & Glo^®^ Reagent to the reaction. Relative luciferase activity was examined as the ratio of firefly/Renilla luciferase activity.

### Statistical Analysis

Data were analyzed with SPSS 19.0 software (SPSS Inc., Chicago, IL, United States) and presented as mean±standard deviation (SD). Student’s t test was used for comparison of two groups. Chi-square test or Fisher’s exact test was used to compare the frequencies of the categorical variables when appropriate. One-way ANOVA was used for comparison among more than two groups. *p*<0.05 was considered as statistically significant.

## Results

### Characterization of the Exosomes

The EVs were derived from each group. The typical saucer-shape hemispherical morphology of EVs was detected by TEM. EV size distribution was measured using TEM and NanoSight technology with the sizes ranging from 30 to 250nm (mean size 125.6nm), which is shown in Supplementary Figures 1A,B. The EV-specific marker CD63 was also detected by western blot analysis in these samples shown in Supplementary Figure 1C. These suggested that the majority of our isolated EVs were exosomes.

### Identification of miRNAs Associated With Plasma Exosomes in Different Groups

**Figure 1:**
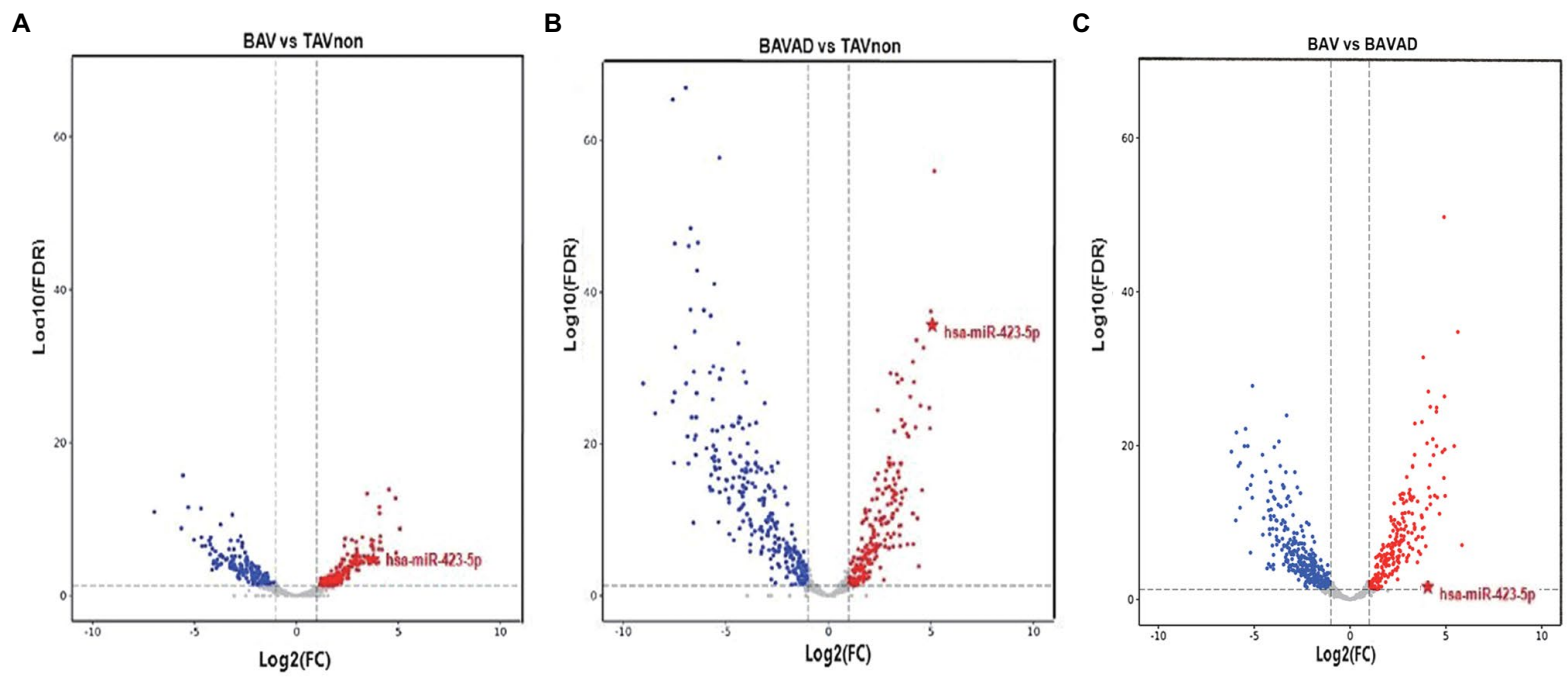
The volcano plot of differential expression levels of the plasma exosome miRNAs between different groups. **(A)** BAV group and TAVnon group. **(B)** BAVAD group and TAVnon group. **(C)** BAV group and BAVAD group. The log_2_ (fold change) is plotted against the −log_10_ (*p* value).

After we filtered and optimized the small RNA sequencing data with FastQC, the modified reads were mapped to various small RNA databases. BAV and BAVAD patients and healthy controls were classified according to miRNA profiles. As shown in volcano plot, the expression levels of miRNAs which were discriminated between TAVnon group and BAV or BAVAD groups, representing the tricuspid or bicuspid morphology of the aortic valve, or between BAVAD group and BAV group, representing BAV patients with or without AAD complication, were, respectively, identified (Figures 1A–C). Accordingly, we identified 174 up-regulated and 154 down-regulated miRNAs in BAV patients compared with healthy individuals, as well as 200 up-regulated and 261 down-regulated miRNAs in BAVAD patients compared with healthy individuals. The top 20 DE miRNAs were shown in Supplementary Materials S2–S4.

### Validation of the Expression of DE miRNAs by RT-QPCR in an Independent Cohort

**Figure 2:**
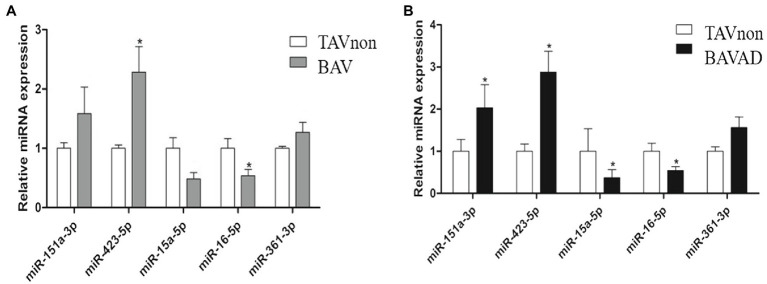
Validation of the candidate DE miRNAs. The selected miRNAs were validated by comparing BAV **(A)** or BAVAD **(B)** patients to healthy TAVnon individuals *via* RT-qPCR. ^*^p<0.05.

Five miRNAs were selected from DE miRNAs according to a previous literature, of which three were up-regulated (miR-151a-3p, miR-423-5p, and miR-361-3p), and two were down-regulated (miR-16-5p and miR-15a-5p) significantly (*p*<0.05). These miRNAs were verified by RT-qPCR from the sequencing results comparing BAV or BAVAD patients to healthy TAVnon individuals, separately (Figures 2A,B). The result showed that these miRNAs went with the same upward or downward trend, although not every miRNA was verified significantly.

### Selection of Candidate DE miRNAs

**Figure 3:**
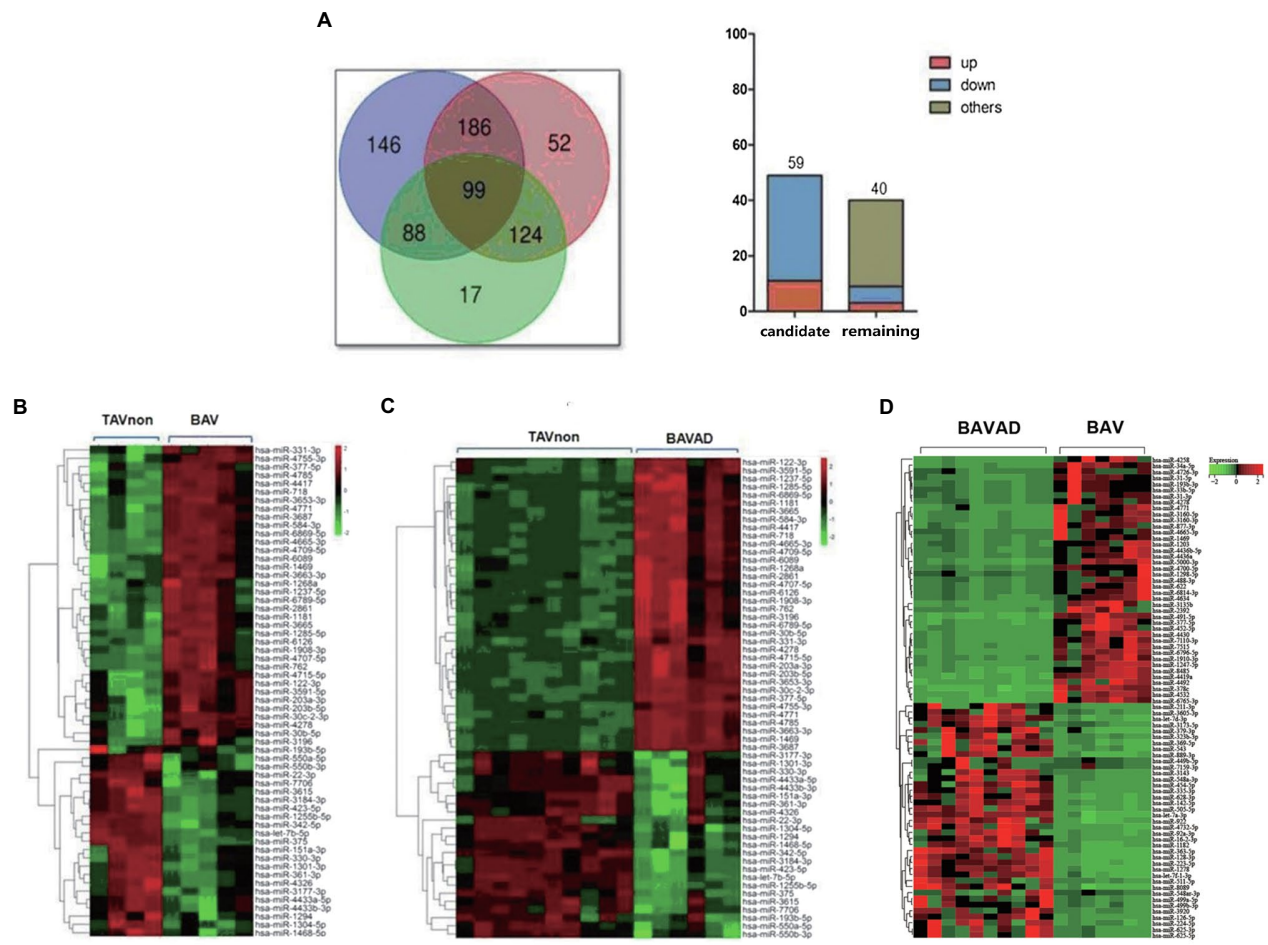
Selection of candidate DE miRNAs. **(A)** Venn diagrams illustrating DE miRNAs between BAVAD vs. BAV (purple), BAVAD vs. TAVnon (red), or BAV vs. TAVnon (green) groups. The upward or downward trend distribution of DE miRNAs among these groups was shown. **(B–D)** Heat map illustrating 59 DE miRNAs between BAV vs. TAVnon group **(B)**, BAVAD vs. TAVnon group **(C)**, and BAV vs. BAVAD group **(D)**. Rows represent miRNAs, while columns represent patients or health individual samples.

First, Venn diagrams were depicted to illustrate DE miRNAs among all three groups. 99 DE exosomal miRNAs were identified in all comparisons (Figure 3A, left). Subsequently, the upward or downward trend distribution of DE miRNAs among three groups was presented. 59 DE miRNAs were defined as candidate miRNAs with the same upward or downward trend in three groups that consisted of literature, of which 11 DE miRNAs were up-regulated, and 48 were down-regulated. These candidate miRNAs were related to the occurrence of BAV disease and the development of BAVAD (Figure 3A, right). Additionally, 9 DE miRNAs with the same upward or downward trend were also associated with the occurrence of BAV or BAVAD disease when comparing BAVAD or BAV patients with TAVnon group, of which 3 DE miRNAs were up-regulated, and 6 were down-regulated (Figure 3A, right). Heat map displayed 59 DE miRNA clusters comparing BAV to TAVnon groups or BAVAD to TAVnon groups or BAV to BAVAD groups (Figures 3B–D).

### Analysis of the Functions of miRNA Target Genes and Integrated miRNA/mRNA/TGF-β Signaling Network

**Figure 4:**
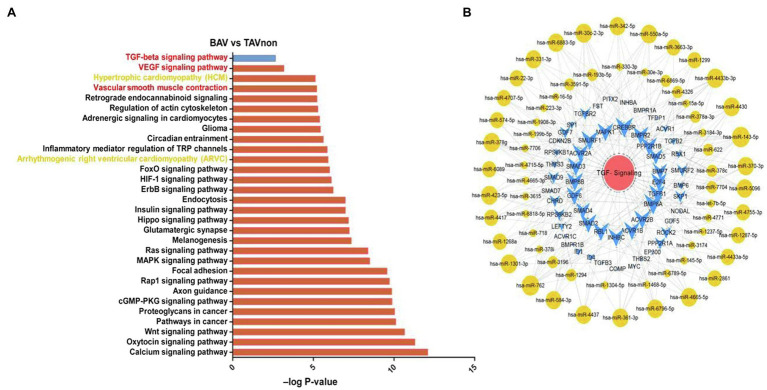
miRNA target gene function analysis and integrated miRNA/mRNA/TGF-β signaling network construction. **(A)** Pathway enrichment analysis highlighted the pathways of intracellular signal transduction deduced by the predicted target genes of DE miRNAs between BAV and TAVnon groups. **(B)** Interaction network of miRNAs and target genes involved in TGF-β signaling pathway.

In order to analyze the differential expression of miRNA target genes between BAV patients and TAVnon individuals, GO and KEGG pathway enrichment analysis were performed (Figure 4A). The target genes of DE miRNAs were mainly enriched in GO terms related to transcription. *Heart development* and *Angiogenesis* terms were indicated to be related to heart diseases including BAV and BAVAD (Hitz et al., 2012; Maleki et al., 2013). MMP2 and MMP9 play an important role in *Heart development* and *Angiogenesis* terms related to bicuspid aortopathy (Derynck and Zhang, 2003; Forte et al., 2017). KEGG pathway enrichment analysis showed that the genes targeted by DE miRNAs were enriched in KEGG terms such as *Hypertrophic cardiomyopathy (HCM)*, *Vascular smooth muscle contraction* and *Arrhythmogenic right ventricular cardiomyopathy (ARVC)*, which were closely related to heart disease (Figure 4A). *VEGF signaling pathway* term, which was related to angiogenesis, also aroused our interest. However, numerous recent studies focused on transforming growth factor β-1 (TGFB1 or TGF-β) signaling pathway and its role in bicuspid aortopathy (Martínez-Micaelo et al., 2017). We used Cytoscape to portray an integrated miRNA/mRNA/TGF-β signaling network, which clearly displayed the mRNAs involved in TGF-β signal pathway and the miRNAs regulating their expression (Figure 4B).

### Validation of miR-423-5p Expression and Its Predicted Function of Regulating TGF-β Signaling

**Figure 5:**
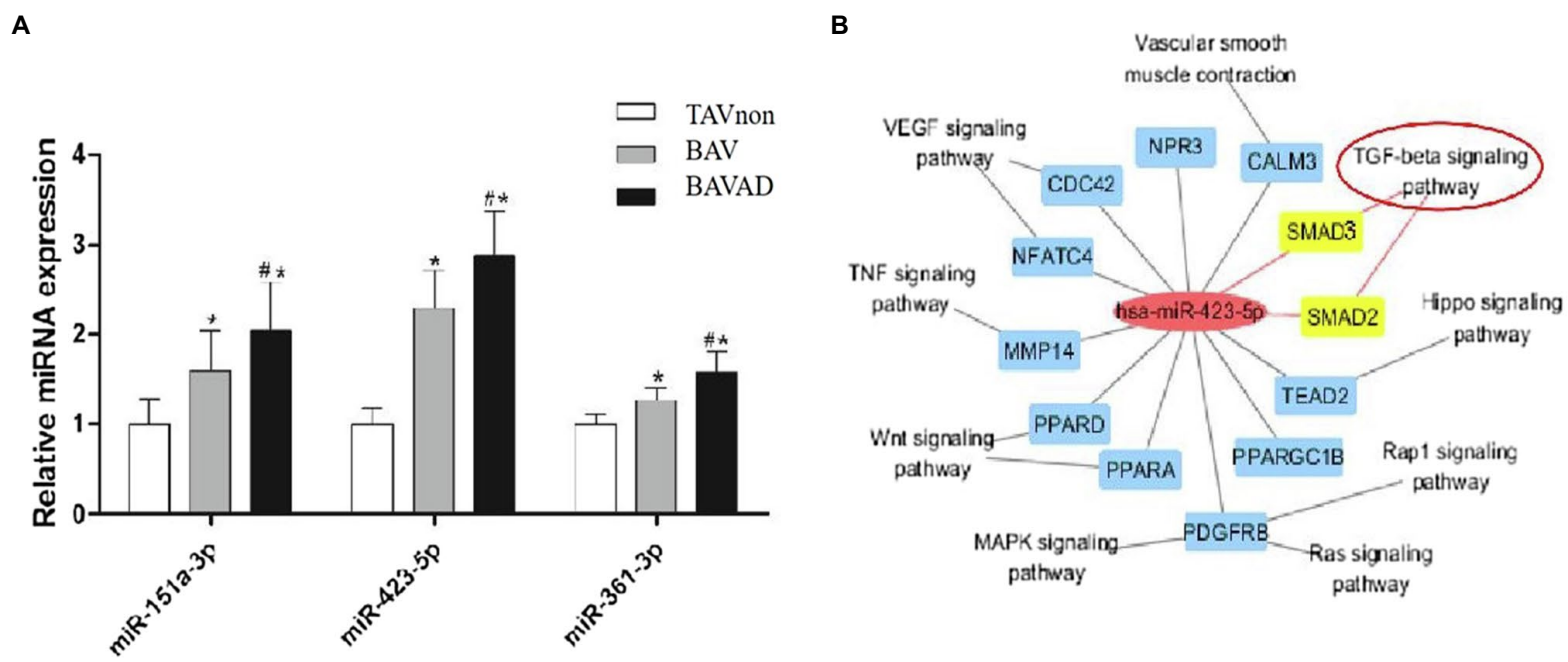
miR-423-5p expression validation and its predicted function of regulating TGF-β signaling. **(A)** The validation of the key miRNA between BAVAD and BAV groups was performed by RT-qPCR. ^*^p (BAVAD or BAV vs. TAVnon)<0.05, ^#^*p* (BAVAD vs. BAV)<0.05; one-way ANOVA. **(B)** Interactive miR-423-5p/mRNA/pathway network. The yellow-marked mRNA is a key target for miR-423-5p to participate in TGF-β signaling pathway.

The expression levels of three DE miRNAs (miR-151a-3p, miR-423-5p, and miR-361-3p), which were significantly increased with the occurrence of BAV disease and the development of BAV into BAVAD, were verified by RT-qPCR (*p*<0.05; Figure 5A). Among them, only miR-423-5p is involved in the regulation of TGF-β signaling. We used Cytoscape to describe an integrated miR-423-5p/mRNA/pathway network, which indicated that miR-423-5p could take part in the occurrence and development of BAV disease through regulating TGF-β signaling, vascular smooth muscle contraction pathway or VEGF signaling pathway (Figure 5B). TGF-β is a multifunctional cytokine which is highly conserved and is involved in various cellular processes, including cell proliferation, differentiation, and apoptosis. Once TGF-β is activated, it binds two subunits of TGF-β type II receptors, then phosphorylates TGF-β type I receptors, which in turn phosphorylate intracellular transduction molecules SMAD2 and SMAD3 (Derynck and Zhang, 2003). Thus, we predicted that the possible mechanism was that miR-423-5p regulated TGF-β signaling by targeting SMAD2/3, and then influencing the occurrence and development of BAV disease and its complications BAVAD.

### miR-423-5p Down-Regulated the Levels of SMAD2 and P-SMAD2 Proteins *in vitro*

**Figure 6:**
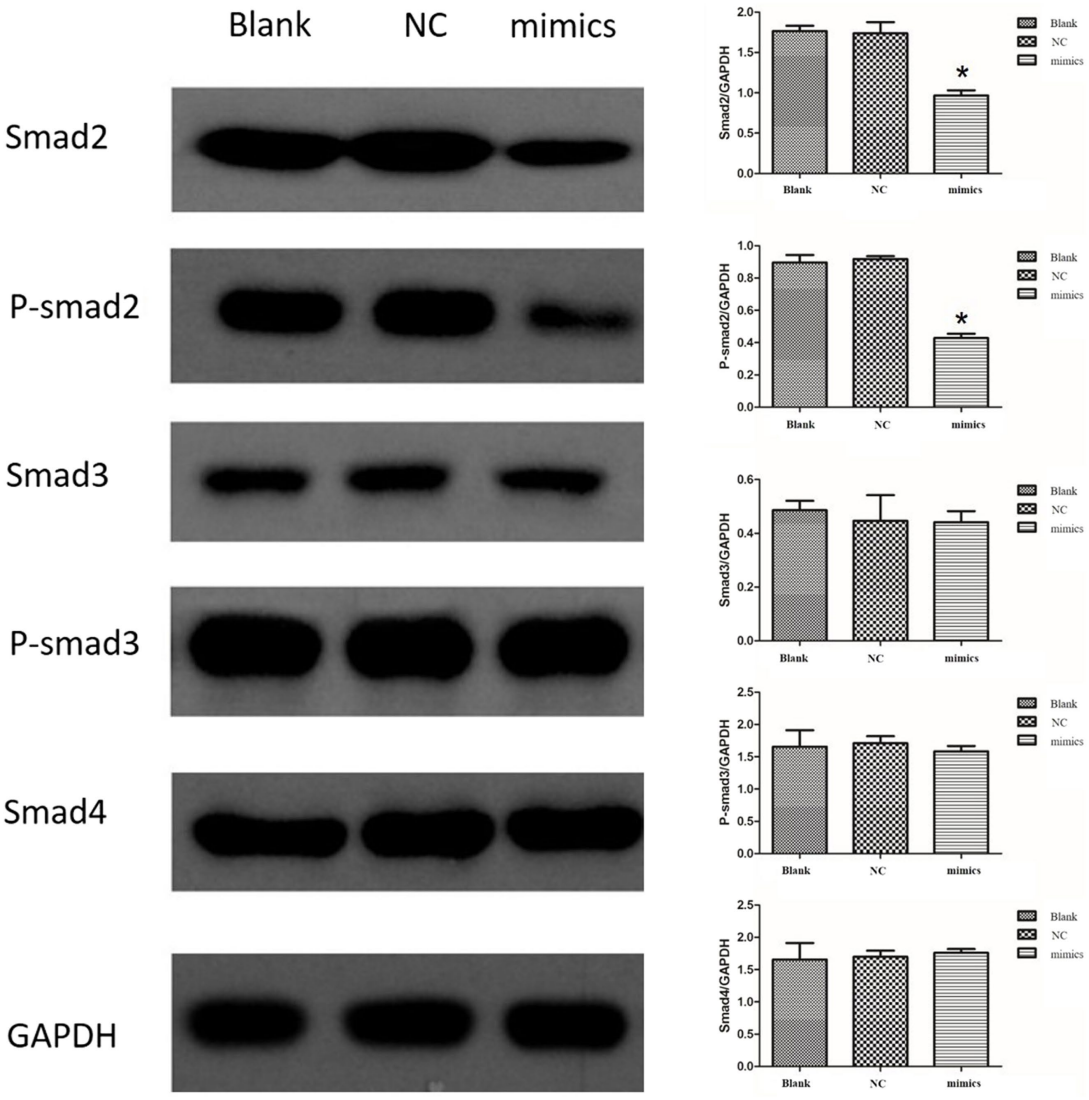
Interaction between miR-423-5p and SMAD2 *in vitro*. The protein levels of SMAD2 and P-SMAD2 were down-regulated by miR-423-5p mimics, while the levels of SMAD3, P-SMAD3, and SMAD4 were not significantly influenced. ^*^p<0.05.

To explore the potential target of miR-423-5p, we detected the protein levels and phosphorylation levels of several crucial members of SMAD family *in vitro*. The levels of SMAD2 and phosphorylated SMAD2 (P-SMAD2) proteins in human aortic VSMCs decreased after the transfection of miR-423-5p mimics, but the levels of SMAD3, P-SMAD3 and SMAD4 proteins did not change significantly. These results suggested that miR-423-5p negatively regulated the expression of SMAD2 and P-SMAD2 proteins in human aortic VSMCs *in vitro* (Figure 6).

### Molecular Interaction Between miR-423-5p and SMAD2 *in vitro*

**Figure 7:**
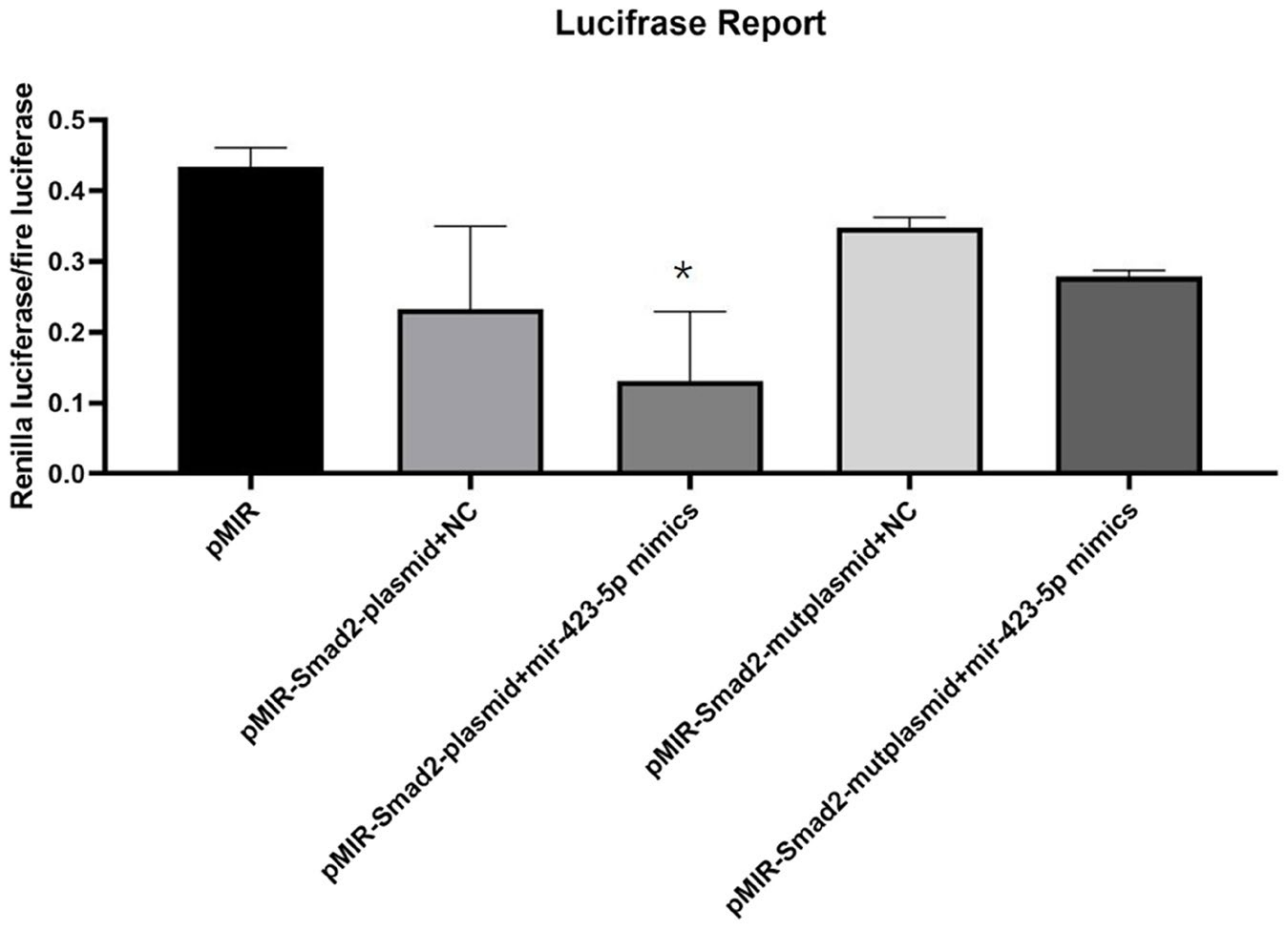
Interaction between miR-423-5p and SMAD2 *in vitro*. Luciferase reporter assay illustrated the luciferase activity of each group, indicating that miR-423-5p could interact with SMAD2 mRNA with the indicated binding site, not the mutant binding site. ^*^p<0.05.

Luciferase reporter assay showed that the luciferase activity of pMIR-SMAD2 plasmids was significantly reduced in human aortic VSMCs transfected with miR-423-5p mimics. However, when human aortic VSMCs were co-transfected with pMIR-SMAD2-mut plasmids and miR-423-5p mimics, the reduction of luciferase activity was significantly attenuated (Figure 7). These results suggested that miR-423-5p could negatively regulate the expression of SMAD2 by binding to the 3'UTR of SMAD2 mRNA at the indicated binding site.

## Discussion

BAV is a common congenital heart malformation accompanied by aortic diseases such as AAD, which significantly increases the risk and cost of surgical treatment, and raises the incidence of postoperative complications and mortality. Therefore, in this study, the pathogenesis of BAV combined with AAD is explored to provide a potential target for early diagnosis and treatment of this disease.

In the analysis of the demographic and clinical characteristics of patient samples, the pairwise comparison between BAV and BAVAD disease groups and TAVnon group showed no difference in *Age*, *Sex*, *Height*, *Weight*, and *Body surface area* indices (Tables 1, 2). Most intriguingly, there was a significant difference between these groups among the index of *Ascending aorta diameter* (^***^p<0.0001)*, Aortic valve gradient* (^*^p=0.0328 or 0.041), *Maximum flow rate* (^*^p=0.0195 or 0.0494), and *People with aortic stenosis* (^***^p=0.000). BAVAD patients all had a large ascending aorta diameter with a lower aortic valve gradient and maximum flow rate when contrasted with BAV and TAVnon samples. This is consistent with hemodynamic theory that the larger the diameter of the aorta, the thinner the vessel wall and the greater the tension of the vessel wall, the easier it is for the ascending aorta to expand and further cause the rupture of aortic dissection. At the same time, the shear force on the anterolateral portion of the ascending aorta was increased in patients with BAV. Additionally, reports revealed that BAV patients were more likely to develop aortic stenosis when compared with aortic insufficiency. This is consistent with our statistical results.

Exosomal miRNAs have been proved to be relevant to the pathogenesis of multiple cardiovascular diseases. Exosomal miRNAs can modulate angiogenesis in endothelial cells, as well as inflammation and fibrillation in cardiomyocytes (Beuzelin and Kaeffer, 2018; Danielson et al., 2018). For BAV aortopathy, there were a lot of investigations of gene or miRNA expression profile among them, many of which revealed specific mRNA or miRNA biomarkers in the disease (Wu et al., 2016; Forte et al., 2017). However, we observed that with the occurrence of BAV disease and its development into BAVAD, the expression of miRNAs showed different tendencies. For example, the expressions of hsa-miR-2,861, hsa-miR-4,785, and hsa-miR-377-5p were high in healthy TAVnon group and moderate in BAV group, while their expressions were low in BAVAD group. Nevertheless, the expressions of hsa-miR-1255b-5p, hsa-miR-3,184-3p, and hsa-miR-423-5p showed the opposite trend. This further indicated that the selected candidate miRNAs could be used as important indicators of disease occurrence and development for further research.

Evidence suggested that BAV patients with aortic dilation showed an increased incidence of cultured VSMC loss. During pathologic vessel remodeling, VSMCs embedded within the collagen-rich matrix infiltrate the subendothelial space and generate neointimal lesions (Johnson and Galis, 2004). When the function of VSMCs is impaired, the synthesis of ECM components is reduced, and the vascular wall structure is damaged, which eventually causes aortic dilation. Interestingly, ascending aortae without dilation of the BAV also have a higher rate of VSMC apoptosis (Nataatmadja et al., 2003). The histological abnormality of BAV and BAVAD disease may arise from a developmental defect of neural crest cells, resulting in premature VSMC apoptosis and abnormal ECM protein transport associated with increased apoptosis of VSMCs in BAV thoracic aortic aneurysm (Nataatmadja et al., 2003). The activity and expression of multiple MMPs in the aorta of BAV patients with AAA were higher than those of normal leaflet aortic aneurysm patients (Ikonomidis et al., 2007). MMP2 and MMP9 have been reported as regulators for the migration of VSMCs. The activity of MMPs is specifically inhibited by forming TIMP-MMP complexes. Among them, TIMP2 is an inhibitor mainly targeting MMP2 and MMP9, which is mainly secreted by VSMCs and fibroblasts and is most commonly expressed in the aorta (Wilton and Jahangiri, 2006). VSMC apoptosis, phenotypic transformation, and high secretion of MMPs lead to arterial vascular structure destruction, increased cell migration, and decreased ECM components, which eventually lead to the occurrence of BAVAD. One of the terms from our GO analysis, *angiogenesis*, and two terms of KEGG pathway analysis, *Vascular smooth muscle contraction* and *VEGF signaling pathway*, were associated with angiogenesis and reconstruction. MMP family members including MMP2 and MMP14 and MAPK signaling members may play an important role in the construction and distension of aorta (Johnson and Galis, 2004). The mechanisms governing ECM degradation and smooth muscle cell (SMC) loss in the ascending aortae of BAV patients are regulated by VEGF (Phillippi et al., 2010). These all provide more possibilities for the mechanism study of exosomal miRNAs in the development of BAV and ascending aortic disease.

TGF-β, a multifunctional cytokine, plays a key role in cell differentiation, apoptosis, and ECM integrity (Meng et al., 2016). TGF-β1 and TGF-β type I and II receptors activate several signaling pathways, such as SMAD-dependent or SMAD-independent pathways, by regulating the expression of ECM proteins, MMPs, and factors of fibrinolytic system (Dong et al., 2002; Derynck and Zhang, 2003). A recent research showed that the ratio of circulating TGF-β1 to soluble endoglin was a possible early biomarker for bicuspid aortopathy (Forte et al., 2017). Enhancement of TGF-β signaling pathway was detected in the aortic dilation patients (Jiao et al., 2016), and the pathway was proved to be required for integrity of the developed vascular by *Tgfbr2* knockout mouse model (Li et al., 2014). Moreover, TGF-β signaling was critical for differentiation and survival of neural crest stem cells (NCSCs)-derived SMCs of BAV patients for maintaining the contractile phenotype late in life and might be the cause of the observed aortopathy in BAV patients with AAA (Jiao et al., 2016). Previous study also found that BAV aorta has lower phosphorylated SMADs levels and availability of free TGF-β compared with TAV which suggested development of bicuspid aortopathy is not monogenic form such as Marfan and Loeys–Dietz syndromes (Paloschi et al., 2015). Recently, a miRNA microarray analysis showed that miR-122, miR-130a, miR-486, and miR-718 were associated with BAV and aortic dilation principally by the activation of TGF-β pathway and vascular remodeling mediated by VEGF signaling pathway (Martínez-Micaelo et al., 2017). In our study, miR-423-5p was found to be involved in heart disease. Targeting miR-423-5p reversed exercise training-induced hyperpolarization-activated cyclic nucleotide-gated potassium channel 4 (HCN4) remodeling and sinus bradycardia (D'Souza et al., 2017). The participation of miR-423-5p in regulating TGF-β signaling in BAV and its complications, however, has not been illustrated yet. We found that the expression of exosomal miR-423-5p was significantly up-regulated not only in BAV group compared with normal TAVnon group, but also in BAVAD group compared with BAV group. In the analysis of miRNA/mRNA/pathway interaction network, miR-423-5p could participate in multiple signaling pathways by targeting different target genes. The most likely mechanism was that miR-423-5p was involved in TGF-β signaling pathway in a SMAD-dependent manner by targeting SMAD2/3, leading to the occurrence of BAV and the development of aortic dilation. To verify the results of target genes prediction *in vitro*, we conducted western blot analysis and luciferase reporter assay in VSMCs. The results suggested that miR-423-5p could negatively regulate the expression of SMAD2 by binding to the 3′UTR of SMAD2 mRNA at the indicated binding site.

To sum up, we innovatively proposed the expression profile of miRNAs in plasma exosomes of BAV patients, as well as the mechanism of miRNAs in regulating the occurrence of BAV and the development of its complication BAVAD. Furthermore, we found a new biomarker for BAV derived from plasma exosomes, miR-423-5p, which was involved in the regulation and progression of BAV disease through targeting SMAD2 to regulate TGF-β signaling. Our result may provide guidance for the diagnosis and treatment of BAV and bicuspid aortopathy. Meanwhile, the limitations of the study are also apparent. Our study is a cross-sectional study, whether patients in the BAV group will have aortic dilatation later in life and the changes in miRNA expression after dilatation require our long-term follow-up study to conclude. Also, the lack of animal models makes it difficult to verify the correlation between serum exosome miRNA expression levels and disease.

## Data Availability Statement

The datasets presented in this study can be found in online repositories. The names of the repository/repositories and accession number(s) can be found at: SRA, PRJNA774820.

## Ethics Statement

The studies involving human participants were reviewed and approved by the Human Research Ethics Committee of Zhongshan Hospital. The patients/participants provided their written informed consent to participate in this study.

## Author Contributions

HZ contributed to conceptualization, data curation, formal analysis, writing—original draft, and writing—review and editing. DL contributed to data curation, software, and writing—original draft. SZ contributed to data curation, formal analysis, and writing—original draft. FW contributed to formal analysis and writing—original draft. XS helped in validation. SY contributed to writing—original draft. CW contributed to conceptualization, validation, and writing—review and editing. All authors agreed to be accountable for the content of the work.

## Conflict of Interest

The authors declare that the research was conducted in the absence of any commercial or financial relationships that could be construed as a potential conflict of interest.

## Publisher’s Note

All claims expressed in this article are solely those of the authors and do not necessarily represent those of their affiliated organizations, or those of the publisher, the editors and the reviewers. Any product that may be evaluated in this article, or claim that may be made by its manufacturer, is not guaranteed or endorsed by the publisher.

## Footnotes

^1^ http://www.bioinformatics.babraham.ac.uk/projects/fastqc/

^2^ http://www.mirbase.org/

^3^ http://www.smallrnagroup.uni-mainz.de/piCdb/

^4^ https://www.ncbi.nlm.nih.gov/

^5^ http://gtrnadb.ucsc.edu/

^6^ http://genome.bioch.virginia.edu/trfdb/

^7^ http://rfam.xfam.org/

## Supplementary Material

The Supplementary Material for this article can be found online at: https://www.frontiersin.org/articles/10.3389/fphys.2021. 759035/full#supplementary-material
