# Supplementary material for: Plasma Exosomal Mir-423-5p Is Involved in the Occurrence and Development of Bicuspid Aortopathy via TGF-β/SMAD2 Pathway
Source: Front Physiol. 2021 Dec 10;12:759035. doi: 10.3389/fphys.2021.759035 (PMC8702998; doi:10.3389/fphys.2021.759035)
Supplement: Supplementary file 1 [file Data_Sheet_1.docx]

| **S1** Primers and sequence for RT-qPCR validation | |
| --- | --- |
| **Primers** | **Sequence** |
| hsa-miR-151a-3p-F | GGCGCAGCTAGACTGAAGCTC |
| hsa-miR-423-5p-F | CATTGAGGGGCAGAGAGCG |
| hsa-miR-29c-5p-F | CGCAGTGACCGATTTCTCCT |
| hsa-miR-30b-5p-F | CGGGCAGTGTAAACATCCTACA |
| hsa-miR-375-3p-F | GCAGTTTGTTCGTTCGGCT |
| hsa-miR-16-5p-F | GGGCAGTAGCAGCACGTAAAT |
| hsa-let-7b-5p-F | CGGGCAGTGAGGTAGTAGGTTG |
| hsa-miR-335-5p-F | CGCAGTCAAGAGCAATAACGA |
| hsa-miR-26a-5p-F | GCGCAGTTCAAGTAATCCAGG |
| hsa-miR-15a-5p-F | GCGCAGTAGCAGCACATAATG |
| hsa-miR-342-5p-F | GGGAGGGGTGCTATCTGTGA |
| hsa-miR-361-3p-F | GCAGTCCCCCAGGTGTGATT |
| miR-R | AGTGCGTGTCGTGGAGTCG |
| U6-F | CGATACAGAGAAGATTAGCATGGC |
| U6-R | AACGCTTCACGAATTTGCGT |

**S2** The top 20 DE miRNAs BAV VS TAVnon

| **ID** | **log2FC** | | **FDR** | **Style** |
| --- | --- | --- | --- | --- |
| hsa-miR-370-3p | 5.07838473 | 1.80E-09 | | up |
| hsa-miR-1299 | 4.893743224 | 2.56E-06 | | up |
| hsa-miR-320a | 4.859892677 | 1.79E-13 | | up |
| hsa-miR-320b | 4.54430747 | 1.36E-14 | | up |
| hsa-miR-1307-3p | 4.205297789 | 9.90E-07 | | up |
| hsa-miR-2110 | 4.118542615 | 1.69E-08 | | up |
| hsa-miR-4665-5p | 4.098700906 | 9.42E-08 | | up |
| hsa-miR-320d | 4.076555769 | 1.64E-11 | | up |
| hsa-miR-320c | 4.068073291 | 2.52E-12 | | up |
| hsa-miR-1247-5p | 4.039653959 | 8.98E-07 | | up |
| hsa-miR-509-3-5p | -4.288736363 | 6.67E-08 | | down |
| hsa-miR-3607-3p | -4.466166789 | 3.14E-07 | | down |
| hsa-miR-1237-5p | -4.634773625 | 3.37E-07 | | down |
| hsa-miR-7111-3p | -4.664145929 | 2.23E-08 | | down |
| hsa-miR-4485-3p | -4.6814832 | 3.91E-12 | | down |
| hsa-miR-1973 | -5.02904573 | 5.31E-08 | | down |
| hsa-miR-615-5p | -5.304048704 | 2.81E-12 | | down |
| hsa-miR-550a-3-5p | -5.572306872 | 2.03E-16 | | down |
| hsa-miR-4262 | -5.648258123 | 1.57E-09 | | down |
| hsa-miR-4495 | -6.975279008 | 1.15E-11 | | down |

| **S3** The top 20 DE miRNAs BAVAD VS TAVnon | | | |
| --- | --- | --- | --- |
| **ID** | **log2FC** | **FDR** | **Style** |
| hsa-let-7b-5p | 5.167069 | 1.20E-56 | up |
| hsa-miR-3184-3p | 5.010831 | 1.80E-36 | up |
| hsa-miR-423-5p | 4.99193 | 3.58E-38 | up |
| hsa-miR-342-5p | 4.955884 | 9.20E-23 | up |
| hsa-miR-543 | 4.926774 | 1.93E-25 | up |
| hsa-miR-16-2-3p | 4.634426 | 2.17E-33 | up |
| hsa-miR-511-5p | 4.572118 | 1.28E-14 | up |
| hsa-miR-128-3p | 4.489625 | 9.07E-26 | up |
| hsa-miR-548ar-3p | 4.407988 | 0.0001365 | up |
| hsa-miR-363-5p | 4.344719 | 6.46E-11 | up |
| hsa-miR-1973 | -6.92696 | 1.12E-28 | down |
| hsa-miR-4700-5p | -6.94365 | 1.21E-67 | down |
| hsa-miR-7111-3p | -7.46501 | 1.87E-33 | down |
| hsa-miR-1292-3p | -7.48334 | 4.07E-47 | down |
| hsa-miR-4485-3p | -7.49069 | 1.68E-27 | down |
| hsa-miR-203b-3p | -7.51936 | 3.23E-18 | down |
| hsa-miR-4665-3p | -7.57697 | 4.50E-66 | down |
| hsa-miR-4278 | -7.5822 | 2.42E-26 | down |
| hsa-miR-4495 | -8.43771 | 1.01E-24 | down |
| hsa-miR-3607-3p | -9.02395 | 1.14E-28 | down |

**S4** The top 20 DE miRNAs BAVAD VS BAV

| **ID** | **log2FC** | **FDR** | **Style** |
| --- | --- | --- | --- |
| hsa-miR-548ar-3p | 5.83703 | 8.55E-08 | up |
| hsa-miR-628-3p | 5.62427 | 1.67E-35 | up |
| hsa-miR-454-5p | 5.43400 | 1.13E-20 | up |
| hsa-miR-3143 | 4.95963 | 3.43E-14 | up |
| hsa-miR-625-3p | 4.93984 | 3.23E-20 | up |
| hsa-miR-335-3p | 4.92445 | 4.04E-27 | up |
| hsa-miR-128-3p | 4.90411 | 1.93E-50 | up |
| hsa-miR-1278 | 4.89774 | 1.60E-16 | up |
| hsa-miR-625-5p | 4.81482 | 6.80E-20 | up |
| hsa-miR-3920 | 4.66476 | 7.27E-12 | up |
| hsa-miR-6796-5p | -5.33673 | 1.13E-20 | down |
| hsa-miR-1292-3p | -5.36791 | 4.05E-15 | down |
| hsa-miR-6883-5p | -5.44996 | 6.50E-23 | down |
| hsa-miR-1273e | -5.51037 | 1.15E-20 | down |
| hsa-miR-6872-3p | -5.73196 | 1.78E-18 | down |
| hsa-miR-7156-5p | -5.73900 | 1.18E-12 | down |
| hsa-miR-1909-3p | -5.82840 | 4.38E-18 | down |
| hsa-miR-6818-5p | -5.92981 | 1.94E-22 | down |
| hsa-miR-3192-3p | -5.96165 | 5.28E-11 | down |
| hsa-miR-3611 | -6.19055 | 6.03E-20 | down |
