# Supplementary figures and images for: Plasma Exosomal Mir-423-5p Is Involved in the Occurrence and Development of Bicuspid Aortopathy via TGF-β/SMAD2 Pathway
Source: Front Physiol. 2021 Dec 10;12:759035. doi: 10.3389/fphys.2021.759035 (PMC8702998; doi:10.3389/fphys.2021.759035)

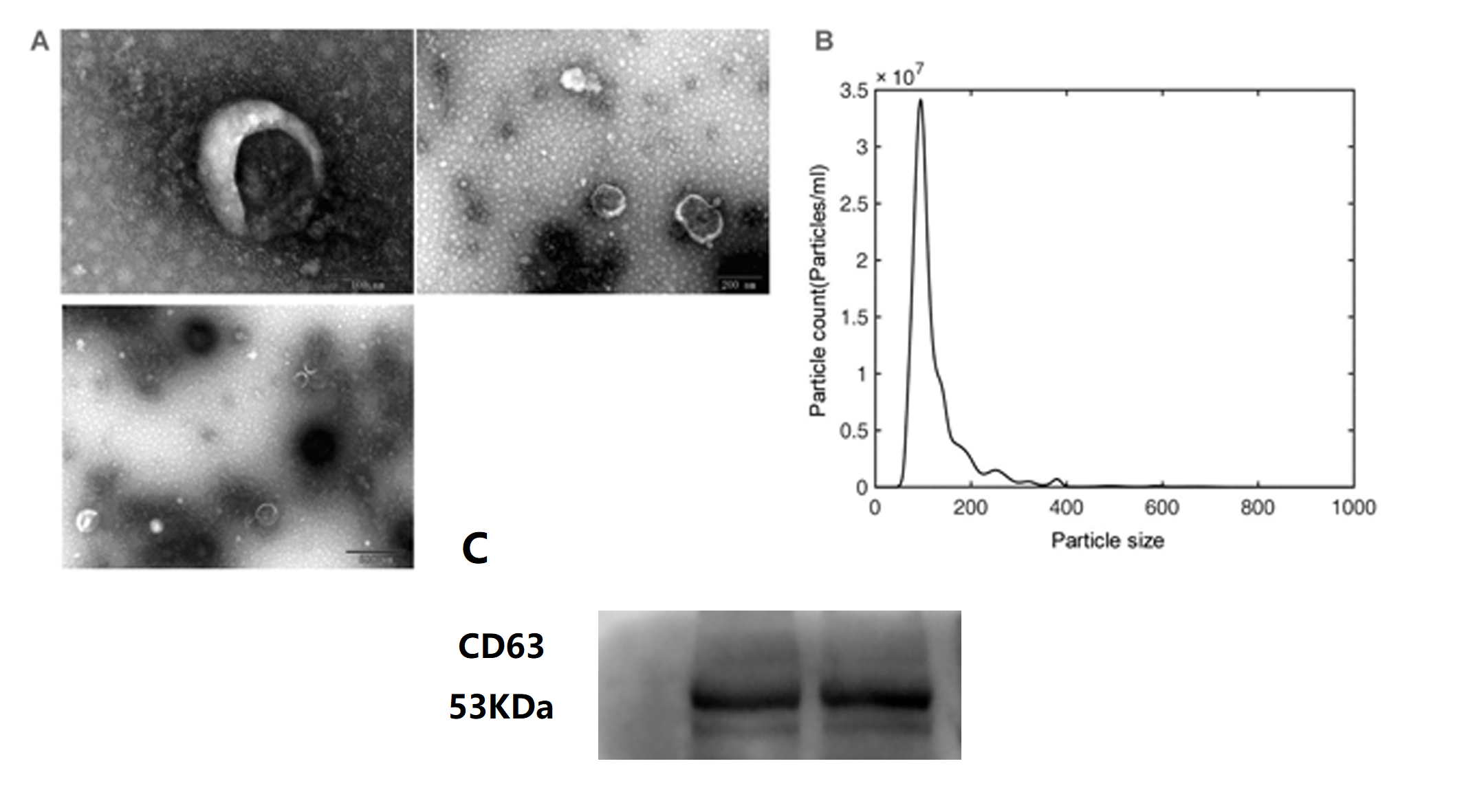

Supplement: Supplementary file 2 [file Image_1.TIF]
